# Acute Inhibitory Effects of Antidepressants on Lacrimal Gland Secretion in the Anesthetized Rat

**DOI:** 10.1167/iovs.62.7.8

**Published:** 2021-06-07

**Authors:** Martin Dankis, Ozgu Aydogdu, Gunnar Tobin, Michael Winder

**Affiliations:** 1 Department of Pharmacology, Institute of Neuroscience and Physiology, the Sahlgrenska Academy, University of Gothenburg, Gothenburg, Sweden

**Keywords:** tear secretion, lacrimal gland, dry eyes, antidepressant, SSRI, TCA, in vivo, rat

## Abstract

**Purpose:**

Patients that medicate with antidepressants commonly report dryness of eyes. The cause is often attributed to the anticholinergic properties of the drugs. However, regulation of tear production includes a substantial reflex-evoked component and is regulated via distinct centers in the brain. Further, the anticholinergic component varies greatly among antidepressants with different mechanisms of action. In the current study it was wondered if acute administration of antidepressants can disturb production of tears by affecting the afferent and/or central pathway.

**Methods:**

Tear production was examined in vivo in anesthetized rats in the presence or absence of the tricyclic antidepressant (TCA) clomipramine or the selective serotonin reuptake inhibitor (SSRI) escitalopram. The reflex-evoked production of tears was measured by challenging the surface of the eye with menthol (0.1 mM) and cholinergic regulation was examined by intravenous injection with the nonselective muscarinic agonist methacholine (1–5 µg/kg).

**Results:**

Acute administration of clomipramine significantly attenuated both reflex-evoked and methacholine-induced tear production. However, escitalopram only attenuated reflex-evoked tear production, while methacholine-induced production of tears remained unaffected.

**Conclusions:**

This study shows that antidepressants with different mechanisms of action can impair tear production by attenuating reflex-evoked signaling. Further, antimuscarinic actions are verified as a likely cause of lacrimal gland hyposecretion in regard to clomipramine but not escitalopram. Future studies on antidepressants with different selectivity profiles and mechanisms of action are required to further elucidate the mechanisms by which antidepressants affect tear production.

Production of tears is a composite process that involves not only the lacrimal glands but also meibomian glands and goblet cells. The main function of the lacrimal glands is to produce the aqueous component of tears, while meibomian glands and goblet cells produce the lipid and mucin components, respectively. Dry eye disease (DED), also known as dry eye syndrome (DES) or keratoconjunctivitis sicca (KCS), is defined as “*a multifactorial disease of the ocular surface characterized by a loss of homeostasis of the tear film, and accompanied by ocular symptoms, in which tear film instability and hyperosmolarity, ocular surface inflammation and damage, and neurosensory abnormalities play etiologic roles*”.^1^ For patients, the most noticeable symptoms are ocular discomfort and pain. While meibomian gland dysfunction can lead to dry eyes due to rapid evaporation, DED is most commonly associated with lacrimal gland failure leading to decreased production of the aqueous tear component.^1^ Aqueous-deficient DED can arise due to lacrimal gland inflammation, for which treatment with topical anti-inflammatory drugs has been shown to be effective, but can also be caused by contact lens use, Sjögren's syndrome, or a number of pharmacological agents.^2^ The global prevalence of DED is estimated to be around 12–21%.^2^^–^^5^ Among the most frequently reported drugs causing symptoms of dry eyes are antidepressants.^6^^–^^8^ Pharmacological treatment with antidepressants is common in developed countries, with an estimated 10% to 13% of the adult population currently on antidepressant medication.^9^^,^^10^

The xerogenic and xerophthalmic effects of antidepressants, especially TCAs, are often attributed to their antimuscarinic effects.^11^^–^^13^ This notion is often strengthened by studies on antimuscarinic drugs in which patients frequently report both dryness of mouth and eyes.^14^ However, it is possible that inhibitory effects can occur at other levels as well, as has been shown in studies on the production of saliva.^15^^,^^16^ Notably, the lacrimal gland and the submandibular salivary glands are in part regulated by a common center in the brain stem, the superior salivary nucleus.^17^ Corneal afferent nerves, mainly of the ophthalmic branch of the trigeminal nerve, can respond to temperature, chemical, and mechanical stimuli and are the first link in the neural reflex arc for regulation of tear secretion.^18^ If their function is compromised, for instance, by autoimmune-induced inflammation (i.e., Sjögren's syndrome), eye-specific diseases which lead to nerve loss, or as a side effect of drug treatment, tear production is decreased.^19^ The facial nerve (cranial nerve VII) contains efferent autonomic nerve fibers that stimulate tear secretion, and it has been shown that both parasympathetic and sympathetic branches participate in the production of tears.^18^ It is well known that cholinergic agonists cause production of tears, and expression of muscarinic receptors, mainly of the M3 subtype, has been shown in lacrimal and goblet secretory cells as well as on myoepithelial cells.^20^ Meanwhile, the sympathetic secretory response is suggested to be mediated mainly via α_1_-adrenoceptors.^21^ Further, both VIPergic, nitrergic, and purinergic stimuli may contribute to the secretory responses.^22^^,^^23^

The treatment options for DED are few, especially pharmacologically active substances. Patients are instead most often referred to environment modification, topical ocular lubricants, or surgery. This highlights the need for new therapeutic alternatives. However, for that to materialize, greater knowledge is needed regarding the causes for dysregulation of tear production. Considering the vast usage of antidepressants, it is of great interest to outline their effect on tear production in valid animal models. In the current study, with a pharmacological approach of drug effects on tear secretion, it was wondered how acute administration of antidepressants of different types (SSRI, TCA) affect reflex-evoked lacrimal gland secretion. Further, the cholinergic component of tear production, often attributed as the cause for DED in patients treated with antidepressants, was examined. For this purpose, reflex-evoked and methacholine-induced tear production was measured in anesthetized rats before and after a single intravenous injection with either the selective serotonin reuptake inhibitor escitalopram, the tricyclic antidepressant clomipramine or saline, serving as control. The reflex-evoked response was examined by direct application of menthol on the ocular surface.

## Methods and Materials

### Study Design

The current study in which 18 adult male rats of the Sprague-Dawley strain were used (body weight: 275–515 g, average 390 g) was approved by the local ethics committee at the University of Gothenburg (permit 1796/18). The study design and experimentation followed local rules and regulations at the University of Gothenburg as well as the ARVO guidelines for Use of Animals in Ophthalmic and Vision Research. The number of animals were chosen to ensure obtainment of objective and reliable results, that is, avoid underpowering, but at the same time minimize the number of animals used, that is, avoid overpowering.

**Figure 1.**
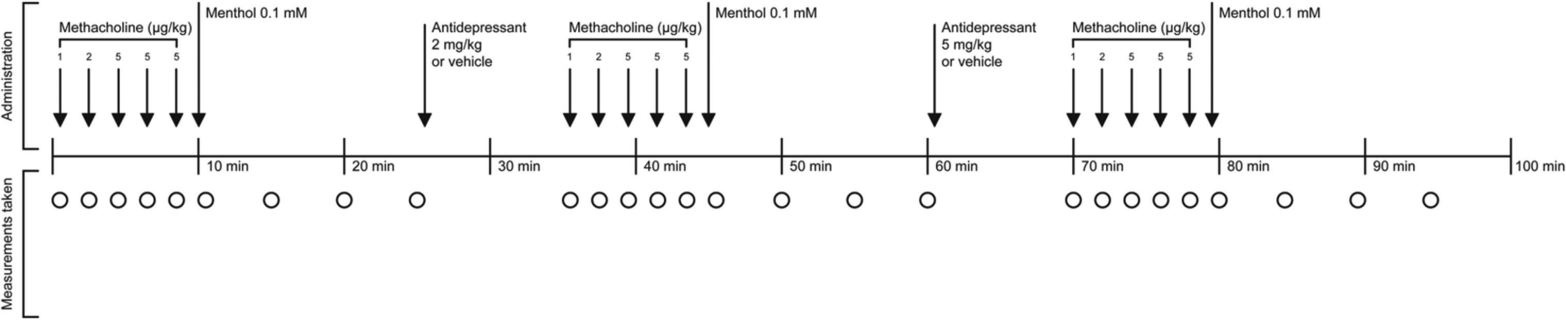
**Schematic description of the experimental procedure.** In a first round of measurements, methacholine was administered in increasing doses (1, 2, and 5 µg/kg i.v.). The highest dose (5 µg/kg) was administered in triplicates and measurements of tear production were taken after each administration. Subsequently, reflex-evoked responses were measured by corneal application of menthol (0.1 mM). Measurements of tear production were taken 0.5, 5, 10, and 15 minutes post application. In the following rounds, measurements of cholinergic- and reflex-evoked tear production were conducted 10 minutes after administration of antidepressant (clomipramine or escitalopram; 2 and 5 mg/kg i.v., respectively) or vehicle (saline; 1 mL/kg i.v.).

The animals were randomized into three groups in which measurements of tear production were performed before and after intravenous administration of either saline (1 mL/kg i.v.; serving as control), the tricyclic antidepressant (TCA) clomipramine (2 and 5 mg/kg i.v.), or the selective serotonin reuptake inhibitor (SSRI) escitalopram (2 and 5 mg/kg i.v.), respectively (Fig. 1; *n* = 6 in each group). The doses of the antidepressants were chosen based on previous studies.^15^ The experimental procedure was initiated by rapid induction of deep anesthesia with a single dose of pentobarbitone (Kronans Apotek, Gothenburg, Sweden; 50 mg/kg i.p.), after which the rats were placed on a thermo-regulated heating pad. To allow for intravenous administration of drugs, a catheter was surgically placed in the femoral vein. To maintain free airways, a cannula was placed in the trachea after performing tracheotomy. Throughout the experiments, anesthesia was maintained by small doses (1–5 mg/kg) of pentobarbitone administered intravenously. It should be noted that high doses of pentobarbitone can attenuate nerve responses.^15^^,^^24^ Thus, for valid results when examining reflex-evoked tear production, it was of utmost importance to maintain anesthesia at a minimal level. In accordance with previous studies on reflex-induced secretion responses, to ensure that all experiments were carried out at a proper level of anesthesia, citric acid (0.5 M) was administered onto the tongue before each measurement of tear production.^25^ Induction of a visible reflexive response was considered an indication for the examination to be commenced. Absence of a visible reflexive response was considered an indication for pentobarbitone-induced attenuation of signal transduction. In those instances, a new test was carried out every 10 minutes until a reflexive response occurred.

### Cholinergic-Evoked (Parasympathetic) Tear Production

Cholinergic induction of tear production was examined by intravenous administration of the nonselective muscarinic agonist methacholine. Pilot experiments were conducted to determine the suitable doses of methacholine (1, 2, and 5 µg/kg, respectively). After administration of each dose of methacholine, tear production was measured by gently placing a preweighed filter paper on the corneal surface and allowing absorption for 30 seconds. Subsequently, each strip of filter paper was weighed and the corresponding increase in weight was noted. The density of the absorbed fluid was considered to be 1 g/mL and therefore recalculated to a corresponding volume, that is, 1 µL per µg.

### Reflex-Evoked Tear Production

Reflex-evoked, centrally mediated tear production was examined by application of menthol to the corneal surface (0.1 mM). Application of menthol to the corneal surface causes mild cooling, evoking reflex-induced production of tears.^26^ In the current protocol, 10 µL of a mixture of artificial tears (106.5 mM sodium chloride, 26.1 mM sodium bicarbonate, 18.7 mM potassium chloride, 1 mM magnesium chloride, 0.5 mM sodium phosphate, 1.1 mM calcium chloride, and 10 mM HEPES) containing 0.1 mM menthol with an adjusted pH of 7.45 was applied directly onto the corneal surface, similar to a previous study.^27^ This caused a blinking reflex and after 30 seconds the existing tear volume was absorbed by filter paper and discarded. Thereafter, a 15 minute period ensued during which the produced tear volume was absorbed by preweighed strips of filter paper every five minutes. Subsequently, each strip of filter paper was weighed and the corresponding increase in weight was noted. Again, the density of the absorbed fluid was considered to be 1 g/mL and the total weight during the 15 minute period of menthol application was recalculated to a corresponding volume, that is, 1 µL per µg. To confirm that the reflex was induced by menthol, pilot experiments were performed in which the tear production was measured in response to application of artificial tears in the absence of menthol. At the end of each experiment the rats were intravenously injected with an overdose of pentobarbitone. After a few minutes, the heart was cut to ensure euthanasia.

### Drugs

The drugs employed were menthol, acetyl-β-methacholine chloride (methacholine; MeCh; nonselective muscarinic agonist), escitalopram oxalate (selective serotonin reuptake inhibitor; SSRI), and clomipramine hydrochloride (tricyclic antidepressant; TCA). All drugs were purchased from Sigma-Aldrich, St Louis, MO, USA.

### Statistical Calculations

Statistical significance was determined by two-way analysis of variance (ANOVA) followed by a Dunnett's test for multiple comparisons. *P* values < 0.05 were regarded as statistically significant. All values are expressed as mean ± SEM. Statistics and graphs, including curve fitting with nonlinear regression, were generated and parameters computed using GraphPad Prism 8 (GraphPad Software Inc., San Diego, CA, USA).

## Results

**Figure 2.**
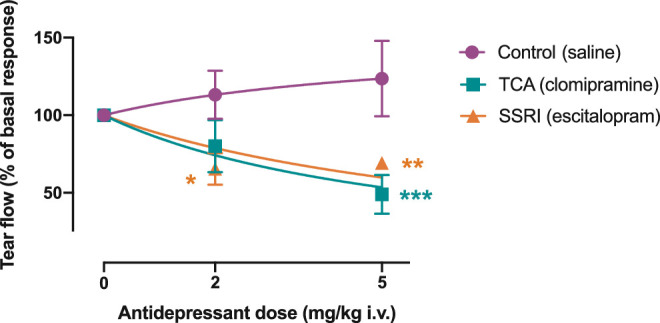
**Reflex-evoked tear production.** Tear production was measured in response to application of 0.1 mM menthol to the cornea over a period of 15 minutes. The volume was measured in the absence (basal response) or presence of antidepressants (escitalopram, SSRI; clomipramine, TCA) in increasing doses (2 and 5 mg/kg i.v.). In control experiments, menthol was administered repeatedly (three times) in the absence of antidepressants. The production upon the first administration was considered the basal response. A two-way ANOVA followed by Dunnett's post hoc test was used for statistical comparison. *, **, and *** denote significant difference (*P* < 0.05, < 0.01, and < 0.001, respectively) between control and treatment group; *n* = 6.

Application of menthol to the corneal surface evoked production of tears throughout the 15 minute procedure. In controls (*n* = 6), this led to an average tear production of 0.39 ± 0.06 µL/min over the 15 minute period. Repeated exposure to menthol caused a slight, but not significant, increase of tear production in controls (by +13 ± 16% and +24 ± 24% in the second and third cycle, respectively; Fig. 2). In rats that received an intravenous injection of escitalopram (*n* = 6), the production was significantly decreased (−34.6 ± 10.3% at a dose of 2 mg/kg, *P* = 0.018; −30.8 ± 3.8% at a dose of 5 mg/kg, *P* = 0.0065; Fig. 2). Likewise, in rats that received an intravenous injection of clomipramine (*n* = 6), tear production was significantly decreased at a dose of 5 mg/kg (−19.9.6 ± 16.8% at a dose of 2 mg/kg, *P* = 0.12; −51.0 ± 12.5% at a dose of 5 mg/kg, *P* < 0.001; Fig. 2). Application of artificial tears in the absence of menthol did not lead to any substantial induction of tear production (0.06 ± 0.04 µl/min over the 15 minute period; *n* = 2; data not shown).

**Figure 3.**
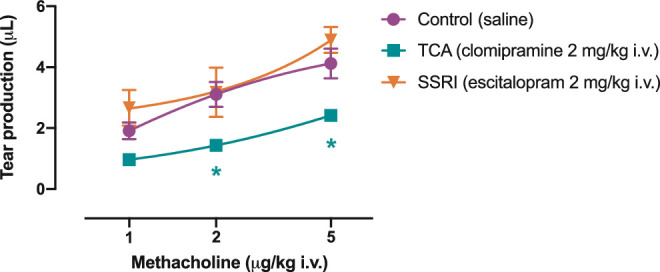
**Methacholine-evoked tear production.** The composite response leading to production of tears was measured in relation to increasing doses methacholine (1, 2, and 5 mg/kg) in the absence (control) and presence of antidepressants (2 mg/kg i.v.; escitalopram, SSRI; clomipramine, TCA). A two-way ANOVA followed by Dunnett's post hoc test was used for statistical comparison. * denotes significant difference (*P* < 0.05) between control and clomipramine; *n* = 6.

Intravenous administration of methacholine led to production of tears in a dose-dependent manner. In controls, tear production was 1.91 ± 0.27 µL/min, 3.11 ± 0.41 µL/min, and 4.12 ± 0.49 µL/min at 1, 2, and 5 µg/kg methacholine, respectively (Fig. 3). In rats that received an intravenous injection of clomipramine (2 mg/kg), the production was significantly decreased in response to 2 and 5 µg/kg methacholine, respectively (from 3.11 ± 0.41 to 1.43 ± 0.20 µL/min, *P* = 0.041, and from 4.12 ± 0.49 to 2.42 ± 0.10 µL/min, *P* = 0.037, respectively; Fig. 3). The production was not further reduced when rats were given a higher dose of clomipramine (5 mg/kg; 1.80 ± 0.13 µL/min and 2.55 ± 0.16 µL/min at 2 and 5 µg/kg methacholine, respectively; data not shown). Contrarily, in rats that were administered escitalopram (2 mg/kg), tear production in response to methacholine was unaffected (2.67 ± 0.59 µL/min, 3.18 ± 0.81 µL/min, and 4.89 ± 0.42 µL/min at 1, 2, and 5 µg/kg methacholine, respectively; Fig 3). Tear production was similarly unaffected by administration of a higher dose of escitalopram (5 mg/kg; 2.40 ± 0.67 µL/min, 2.83 ± 0.48 µL/min, and 4.57 ± 0.59 µL/min at 1, 2, and 5 µg/kg methacholine, respectively; data not shown).

## Discussion

The present study shows that reflex-induced tear production is attenuated by acute administration of antidepressants, regardless of their selectivity profile. At the same time, clomipramine, but not escitalopram, attenuates methacholine-induced tear production. Therefore, similar to what was seen in a previous study on the effects of antidepressants on production of saliva,^15^ the antimuscarinic properties of clomipramine are important but cannot alone explain the inhibition of tear production caused by all antidepressants. Instead one must assume that antidepressants also exert attenuation of tear production by directly or indirectly affecting the actions of other signaling molecules, for instance, ATP acting via purinergic receptors^22^ or prostaglandins.^28^ Also, at least regarding clomipramine and considering that systemic beta-blockers are commonly associated with worsening of symptoms of DED,^29^ involvement of the sympathetic nervous system cannot be ignored. Nevertheless, the attenuating effects of the selected antidepressants, especially on nerve-evoked responses, coincide well with the relatively few studies that have examined similar effects in other glands.^15^ In the current study, clomipramine and escitalopram were chosen as representative drugs for SSRIs and TCAs, respectively. The choice was partly based on the possibility to allow for comparisons with previous studies. However, it is important to keep in mind that the choice of drug could play a potential role in the current study. Clomipramine is well known for its antimuscarinic properties.^11^ Contrarily, escitalopram has less affinity for muscarinic receptors, even compared to other SSRIs.^30^ Likewise, the affinity for other receptors of interest varies greatly among antidepressants. Future in vivo studies should be aimed at further pinpointing the mechanism of attenuation of reflex-evoked tear production, preferably including antidepressants with different selectivity profiles and different mechanisms of action (i.e., SNRIs). Future studies should also investigate the long-term effects of antidepressants on tear production as these may vary from those seen in the current study.

There are striking similarities regarding the effects of antidepressants on production of tears and production of saliva. Foremost are the similar attenuating effects of drugs with different selectivity profiles on reflex-evoked and methacholine-induced production of saliva and tears, respectively.^15^ In this context it is interesting to note that both submandibular salivary glands and lacrimal glands are partly regulated via a common nucleus.^17^ In a previous publication it was noted that the attenuating effects of amphetamine on reflex-evoked production of saliva could be abolished by a combination of α-adrenergic and dopaminergic antagonists.^16^ The authors drew the conclusion that this indicates that amphetamine acts via a central mechanism, rather than a peripheral. Considering this finding and other similarities between salivary glands and the lacrimal gland, including a shared nucleus, a similar possibility to affect reflex-evoked tear secretion via a distinctly central mechanism is likely. The analogous effects of the currently investigated antidepressants on production of saliva and tears are therefore not surprising, but rather expected.

The tear volume that is measured is a composite product. Not only the lacrimal gland but also meibomian glands and goblet cells are involved and contribute to the total volume. In particular, regarding the lacrimal component, glandular blood flow is considered an important factor.^31^ It was noted during the experiments that tears produced upon stimulation with methacholine contained a small amount of blood (haemolacria). This was not visible during reflex-evoked tear secretion. Haemolacria can be observed in humans upon challenge with a parasympathomimetic drug.^32^ Even though haemolacria in humans can be the result of increased blood pressure, here that is unlikely since muscarinic agonists, on the contrary, cause a drop in blood pressure. However, in studies on blood vessels in the rat submandibular gland, it has been shown that muscarinic agonists cause relaxation of arteries but contraction of veins.^33^ This was suggested to lead to an increase in glandular hydrostatic pressure, which could be an explanation for the currently observed heamolacria. Nevertheless, several studies have shown that muscarinic receptors are important for the regulation of glandular blood flow.^34^ Considering the antimuscarinic properties of clomipramine, one can speculate that part of its attenuating effects involve a decrease in blood supply to, mainly, the lacrimal gland.

Using menthol to induce reflex-evoked tear production proved to be robust. Other options exist, for instance, using citric acid or other cooling agents, but all should induce a similar response. The production of tears was maintained at a constant level during the entire 15 minute measuring period, demonstrating that the evoked reflex was maintained despite dilution of the concentration of menthol. Comparably, the total volume of tear production was small during the experiments. Larger tear volumes would be easier to measure, making it easier to perform corresponding studies in larger species. However, despite certain anatomical differences, rodent models are by far the most common for studies on the regulation of tear production. To maintain good precision, tear volume was measured by filter paper absorption. The filter papers were weighed immediately with a high precision analytical balance. An alternative to using filter papers could be the phenol red thread (PRT) test. However, the thin cotton thread used in the PRT test would likely make it challenging to absorb tear volume over time, compared to a broader filter paper. Also, the available literature raises uncertainties regarding the results of the PRT test.^35^^,^^36^ The choice of pentobarbitone for anesthesia is also worth discussing. Pentobarbitone is well known to attenuate reflex-evoked responses.^15^^,^^24^ However, other anesthetics can have similar effects and a majority of previous studies have been performed using pentobarbitone. Further, by keeping anesthesia at a minimal level and testing for valid reflex responses with citric acid before measurement of each reflex-induced response, attenuating effects were currently avoided.

The data in the present study indicate that clomipramine affects the regulation of tear production at all levels (afferent, central, and efferent), while escitalopram mainly affects afferent signaling and/or central pathways. Thus, the current study provides further knowledge of the pharmacological characteristics of antidepressants. However, there is still a need for studies on the long-term effects of antidepressants. It is possible that the currently observed acute hyposecretory effects that resulted from administration of antidepressants may persist during chronic treatment. If so, the absence of interaction between escitalopram and cholinergic stimulation of tear production indicates that muscarinic agonists may be a plausible treatment of hyposecretion due to adverse effects of SSRIs. However, muscarinic agonists, for instance, cevimeline, have already been tested as treatment for dry eyes. Clinical studies have shown that the efficacy of cevimeline is modest, except for patients suffering from milder versions of Sjögren's syndrome.^37^ Thus, there is need for further studies to pinpoint the complex and composite processes that regulate tear production.

## Conclusions

This study shows that a single dose of antidepressants with different mechanisms of action can attenuate reflex-induced tear production. Further, it is shown that the tricyclic antidepressant clomipramine, but not the SSRI escitalopram, attenuates methacholine-induced tear production. Thus, both escitalopram and clomipramine cause hyposecretion, but via partly different mechanisms. Future studies need to be designed to further outline the detailed mechanisms by which antidepressant drugs affect tear production and in which manner this unwanted side effect most effectively can be avoided.
